# Implementation of Numerical Mesostructure Concrete Material Models: A Dot Matrix Method

**DOI:** 10.3390/ma12233835

**Published:** 2019-11-21

**Authors:** Hao Xie, Jili Feng

**Affiliations:** State Key Laboratory for Geomechanics and Deep Underground Engineering, China University of Mining and Technology Beijing, Beijing 100083, China

**Keywords:** mesostructure, aggregates, concrete, parking algorithm, three-dimensional laser scanner

## Abstract

We develop a dot matrix method (DMM) using the principles of computational geometry to place aggregates into matrices for the construction of mesolevel concrete models efficiently and rapidly. The basic idea of the approach is to transform overlap detection between polygons (or polyhedrons) into checking the possibility of any intersection between the point sets within a trial placement aggregate and the already placed ones in mortar. Through the arithmetic operation of integer point sets, the efficiency of the underlying algorithm in the dot matrix method is higher. Our parking algorithm holds several advantages comparing with the conventional placement issues. First, it is suitable for arbitrary-shape aggregate particles. Second, it only needs two sets for examining if the overlap between a trial placement aggregate and the already placed ones. Third, it accurately places aggregates according to aggregate grading curves, by order of reduction, led to more efficiently reducing aggregate placement time. The present method is independent of the size of aggregate particles. Combing with 3D laser scanning technology, the present method can also be used to create mesostructure concrete models conveniently and flexibly. Several examples show that DDM is a robust and valid method to construct mesostructure concrete models.

## 1. Introduction

Concrete is a so-called particulate material on multiscale levels of its constituents. Such constituents crucially influence the properties and performance of concrete. Many investigations have demonstrated that such mesostructure is essential to ensure satisfactory durability and strength in concrete [1,2]. Therefore how to design and optimize the shapes and distribution of fine and coarse aggregate particles in concrete composite models are seriously concerned to understand the macroscopic behaviors of concrete better.

Generally speaking, there are two ways to construct numerical concrete material models in practice. The first one usually uses X-ray computed tomographic (CT) technology to scan concrete specimens section by section. The section imagines captured by such manner can be used to reconstruct the original shapes and distributions of concrete aggregates and mortars under certain image resolution conditions. However, the specimen size is undoubtedly limited, which is usually in the range of the laboratory sample scale [3,4,5]. Similarly, by 3D laser scanning technology, a certain number of grading aggregates are separately photographed, in which the images of individual particles are all stored as a database or library [6,7,8,9]. Then the aggregates taken from the database are randomly placed into a pre-defined container according to a particle grading, to produce numerical composite material models eventually. The placement is in order from the larger ones to the smaller ones. Each new particle that is placed must not overlap any other particles that have already been placed, since that would be unphysical. Indeed, 3D laser scanning technology is much more attractive in use than CT technology. 3D laser scanning is almost harmless. In contrary, X-ray CT is not only expensive in routine use but also has stringent safety and radiation monitoring requirements in practice. Even though the procedures to retrieve particle shape for a given class of aggregates from either CT scanned digital images or 3D laser scanned digital ones have been established [3,7,8,9], it is always appreciated to numerically characterize the shape of particles in terms of good mathematical tools such as spherical harmonic series and Voronoi tessellations [10,11,12,13,14].

In the second one, the generation-and-placement of aggregate particles by the mathematical methods is performed to construct concrete material models, which are perhaps most commonly investigated recently in [3,10,11,13,14,15,16,17]. For example, in the pioneered work, Wittmann et al. [16]. employed the Beddow and Meloy’s morphological law of particles to reconstruct 2D mesostructure concrete composite models [18]. Aggregate particle shapes such as circle and irregular polygon are generated and randomly sequentially added [19]. Subsequently, the concrete meso-level models of particles embedded in matrix materials were developed, in which the regular shape particles are usually like spheres, ellipsoids, and multi-faceted polyhedral [10,20,21]. Generally, the actual complicated aggregate shapes should be taken into account in mesostructured material models. Consequently, spherical harmonic series has been utilized to represent the irregular surfaces of aggregates from the multiscale viewpoint [3,12]. The spherical harmonic approach was further improved and developed in [12,13,22] to more accurately and efficiently generate and park aggregate particles in concrete from the multiscale viewpoint [23]. Of course, successful placement irregular particles randomly into the pre-defined container are a nontrivial task during the implementation of numerical concrete composite material model. Efficiently parking procedures and algorithms are still needed to create the numerical concrete geometrical model with suitable grading particles [24]. Such a placement particle procedure is a usual trial-and-error process of ghost particle positioning, which often costs a lot of Central Processing Unit (CPU) time.

The core issue of placement aggregate procedure is how to detect whether overlapping between aggregates in a pre-determined container efficiently. It is relatively straightforward to realize the detection of overlap between two spherical or elliptical aggregates in the parking process because of the simplicity of their geometry. However, the similar overlap detecting processes are considerably more complicated for irregular particles [10,21,25,26]. For star-shaped particles, Qian and Qian et al. in [13,22] developed a method to examine if two particles overlap or not by solving contact equations. However, the accuracy and efficiency of the algorithms are not very well, and the function is also limited. Recently, Thomas et al. and Garbooczi and Bullard [20,27] improved the overlap algorithm for two 3D irregular particles characterized by the spherical harmonic series. They used the extent overlap box (EOB) to enhance the efficiency of the overlap detect algorithm. The occupation and removal method (ORM) was proposed in [28], to improve the aggregate volume fraction in concrete material models. This approach is suitable to generate the star-shaped aggregate particles. Stroeven et al. and Stroeven et al. [29,30] developed a concurrent algorithm-based discrete element modeling system, HADES, to realistically simulate the packing of irregular-shape particles over the fully compacted state. However, to the best knowledge of the authors, an explicit and universal approach is still missing to place arbitrary-shape aggregates into a pre-defined container and detect overlapping state between the aggregates both efficiently and robustly.

In the present work, we develop a dot matrix method (DMM) that is readily to park arbitrary aggregate particles within matrix based on the principles of computational geometry. The basic idea of DMM is to transform overlap detection between polygons or polyhedra into checking the possibility of any intersection between the point sets within a trial placement aggregate and the already placed ones in matrices. At the same time, we introduce the arithmetic operation of integer point sets. Due to the implementation of the underlying algorithm of such point set operation mainly on integer arithmetic, its code execution speed and efficiency are generally higher. Our parking or placement manner at least holds several advantages comparing with the conventional placement issues. Firstly, it is suitable for arbitrary-shape aggregate particles. Secondly, it only needs two sets for examining if the overlap between a trial placement aggregate and the already placed ones. Thirdly, it accurately places aggregates according to aggregate grading curves, by order of reduction, led to more efficiently reducing aggregate placement time. DMM is especially suitable to construct concrete models with higher aggregate volume fractions. Moreover, DMM is independent of the size of aggregate particles. Thus it can help to investigate and find new properties of multiscale composite models as well as to establish discrete element models of interest. Furthermore, the geometrical features such as volume, area, and inertia tensor, of aggregate particles created in composite material models, are conveniently evaluated and provided due to the use of the discrete scheme of DMM. Combing with either X-ray CT or 3D laser scanning, DMM can be used to create mesostructure concrete models conveniently and flexibly. DMM can also be used to help study reinforced concrete behaviors under complicated loads but this is beyond the scope of the present paper.

The arrangement of the paper is as follows. Section 2 briefly introduces the currently used methods to construct an arbitrary-shape aggregate particle in either 2D case or 3D case. Moreover, we also discuss the widely utilized grading curves to numerically create a database or library of various aggregates in either 2D case or 3D case. In Section 3, the detection methods of a point in polygon or polyhedron are summarized to use in parking aggregates into matrix. A dot matrix method for efficiently creating concrete models is detailed and treated in Section 4. Using this method, we also provide several mesostructured concrete material models to verify and validate its feasibility and robustness in Section 4. Finally, the conclusion is summarized.

## 2. Generation of Aggregates

### 2.1. Description of 2D Aggregate Shape

In the 2D case, Wittmann et al. [16] constructed the geometrical shape of aggregates in concrete based on the methods developed in [18]. Such a method may be in term of a polar coordinate to conveniently represent the position of vertex Pi in a polygon, and its corresponding Cartesian coordinates (*X_i_*, *Y_i_*) are readily obtained through the transformation matrix between two coordinate systems. We will construct aggregates in a similar way to the above work [16] and adequately adjust and improve them to suit our motivation. The procedure is completed by the following three steps.

(1) Generate n polar angles as follows,

(1) $${\bar{\theta }}_{i}=(2\pi /n)[1+(random(-1,1))f_{\theta }],\begin{array}{cc} & i=1,2,\cdots n \end{array}$$

where *n* is the number of sides of the polygon and also the number of vertices of the polygon; $f_{\theta }$ is the angular fluctuation coefficient between 0 and 0.5, generally may take 0.35; random (−1,1) indicates random function that can generate a random number between −1 and 1.

(2) Modify every ${\bar{\theta }}_{i}$ obtained by Equation (1), using Equation (2), so as to gain series $\theta_{i}$,

(2) $${\tilde{\theta }}_{i}={\bar{\theta }}_{i}(2\pi /\sum_{j=1}^{n}{\bar{\theta }}_{j}),\begin{array}{cc} & i=1,2,\cdots \end{array}n$$

(3) $$\theta_{i}=\sum_{j=1}^{i}{\tilde{\theta }}_{j}\begin{array}{cc} & i=1,2,\cdots n \end{array}$$

where $\theta_{i}$ changes between 0 and 2π.

(3) Evaluate the length of $\rho_{i}$ with respect to the polar angle, $\theta_{i}$, as follows

(4) $$\rho_{i}=R[1+(random(-1,1))f_{\rho }]\begin{array}{cc} & i=1,2,\cdots n \end{array}$$

where *R* is the random radius of aggregate; $f_{\rho }$ is the fluctuation coefficient of radius and may change between 0 to 0.5, which generally can take the value of 0.15. Next the polar coordinates of vertices of the polygon, $\left(\rho_{i},\theta_{i}\right)$, can readily be represented by the corresponding Cartesian coordinates, $\left(x_{i},y_{i}\right)$ as follows,

(5) $$x_{i}=\rho_{i}\cos\theta_{i}\begin{array}{cc} & y_{i}=\rho_{i}\sin\theta_{i} \end{array}$$

It is noted that the shape of aggregates can significantly influence not only the porosity of concrete, but also the mechanical behaviors of fresh concrete [1]. The following describes how to improve the aggregate generation scheme described above to make the generated aggregates more realistic.

**Figure 1:**
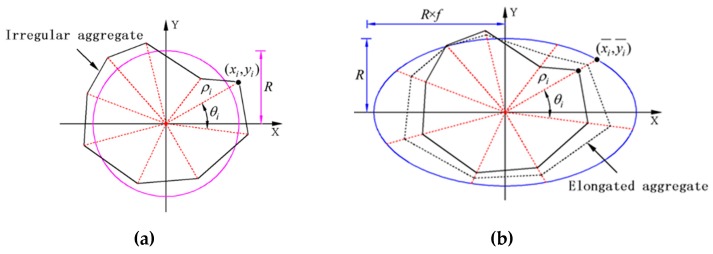
Illustration for generation schemes of (**a**) irregular aggregate and (**b**) elongated aggregate.

The random concavo-convex aggregate is shown in Figure 1a, which is generated by the above process. This method is also the most prevalent generation method of aggregates currently. From Figure 1a, we readily find that the aggregate shape is still with the characteristic of a relative roundness. To make the aggregate shape of numerical concrete closer to the shape of the real aggregate, we will further improve the shape generation scheme as follows.

We first draw an ellipse with a short semi-axis of *R* and a long semi-axis of *R*×*f*, where *f* is the long-short axis scaling factor, which ranges from 1 to 3. The coordinates $\left({\bar{x}}_{i},{\bar{y}}_{i}\right)$ of the intersection of the polar axis with the polar angle $\theta_{i}$ and the ellipse defined are calculated in turn. Then, the coordinates $\left({\tilde{x}}_{i},{\tilde{y}}_{i}\right)$ of the vertices of the stretched polygon can be derived from Equation (6), and the length-to-width ratio *α* of the aggregate after stretching can be approximated by Equation (7). If the coefficient *f* in the range of 1–3 is randomly generated during the programming, the final generated aggregates will exhibit different length-to-width ratios. After doing this, more realistic aggregates can be obtained, as shown in Figure 1b.

(6) $$\left\{ \begin{array}{l} {\tilde{x}}_{i}=(x_{i}+\bar{x})/2\\ {\tilde{y}}_{i}=(y_{i}+{\bar{y}}_{i})/2 \end{array}\right.$$

(7) α≈(R+Rf)/(2R)=(1+f)/2∈[1,2]

### 2.2. Description of 3D Aggregate Shape

In 3D case, the best and fast way is perhaps to employ X-ray CT or 3D laser scanner to gain the morphological description of an individual aggregate particle. If the size of a single particle is not too big, for example, in the range of 10 cm, its morphology and overall 3D distribution of the constituents can be obtained by X-ray CT technology, through scanning one section after another to the studied particles. The cost of X-ray CT scanning is usually high, and the most crucial issue is its safety requirements in applications. However, star-shaped aggregate particles can also be characterized by the spherical harmonic series quantitatively. The similarity is to describe spheres, ellipsoids, and regular polyhedrons by the analytical forms [3,13,20,31].

**Figure 2:**
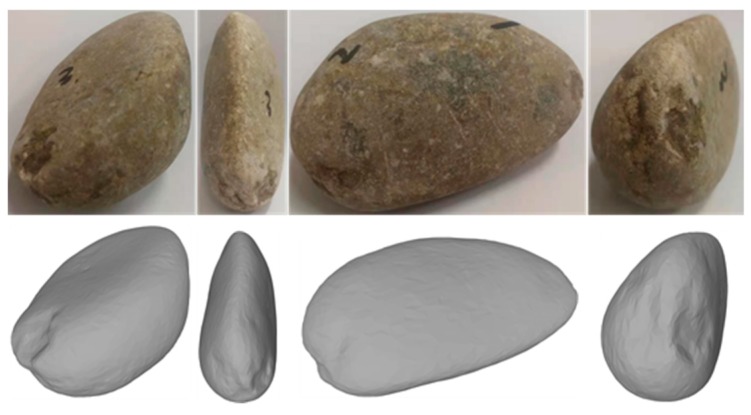
Illustrations for a rounded stone. The upper ones are four photos for the real rounded stone while the lower ones are the corresponding reconstructions by Artec 3D laser scanner.

On the other hand, 3D laser scanning technology is currently in vogue due to its ability to immediately obtain the shape of irregular aggregate particles, both efficiently and accurately. Such the method is almost harmless to living beings in use and is nearly limitless to the size of a scanning particle sample. So, one can employ a 3D laser scanner to gain the realistic morphology of any aggregate particle to create a database or library for generating the concrete composite material models. For example, in Figure 2, the upper is a real rounded stone sample, and the lower is its reconstruction by Artec 3D Laser Scanner [32]. Such images’ resolution is strictly dependent on the accuracy of the 3D laser scanner used, which can be adjusted according to the object scale concerned. For example, the lower resolution of 3D laser scanner may be employed if coarse aggregates were concerned in the study. However, the higher resolution should be used if fine aggregates are investigated.

**Figure 3:**
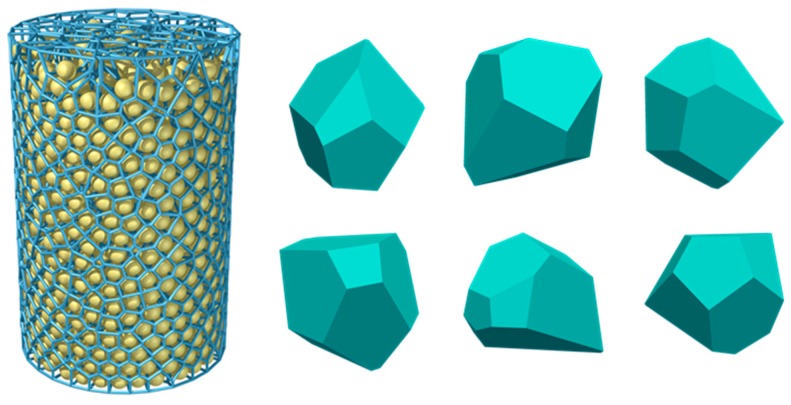
Illustrates some typical polyhedra taken from a 3D Voronoi tessellation for a cylindrical particle packing.

We employed Voro++ to create convex-shaped aggregates [33]. The latter is used to generate concrete composite material models according to grading curves in Section 3. Figure 3 illustrates some typical polyhedra taken from a 3D Voronoi tessellation for a cylindrical particle packing. These polyhedra are also employed to construct the database of concrete composite material models in Section 3 and Section 4.

### 2.3. Construction of Grading Aggregate Database or Library

In the previous section, only the principle of generation of individual aggregates is elaborated. Actual concretes are generally made up of some mixed materials such as cement, sand, and coarse aggregate, to achieve a higher degree of compaction. How to construct numerical concrete material models with an excellent continuous grading of aggregates is a difficult task. At the beginning of the 20th century, Fuller proposed the size distribution curve of aggregates, which is perhaps one of the most commonly accepted 3D ideal grading curves of aggregates at present. It is well known that higher strength and a good density of concrete can be achieved when such a grading curve of aggregates is applied. In 3D case, A Fuller curve is adopted in the following simple form.

(8) $$P(D<D_{i})=100{\left(D_{i}/D_{\max}\right)}^{1/2}$$

where *P*(*D* < *D_i_*) represents the cumulative percentage of weight passing a sieve with diameter *D_i_*, *D_max_* is the largest aggregate size.

Walraven derived the 2D Fuller grading curve of aggregates by the equivalent transformation between 3D distributions and 2D ones of aggregates in concrete under some assumptions of spherical aggregates, which makes the construction of a 2D geometric model of numerical concretes possible to be realized [28]. Ma et al. adopted such the 2D Walraven grading curve to construct concrete material model [28]. We next employ the common thread to construct 2D concrete material models so that the improved Fuller curve is as follows,

(9) $$\begin{array}{ll} P(D<D_{i})= & P_{k}[1.065{\left(D_{i}/D_{\max}\right)}^{0.5}-0.053{\left(D_{i}/D_{\max}\right)}^{4}\\ & \;-0.012{\left(D_{i}/D_{\max}\right)}^{6}-0.0045{\left(D_{i}/D_{\max}\right)}^{8}+0.0025{\left(D_{i}/D_{\max}\right)}^{10}] \end{array}$$

where *P_k_* is the fine and coarse aggregate volume percentage in concrete, which is usually equal to 0.75, *D_i_* is the sieve diameter, *D_max_* represents the maximum diameter of aggregates. For a concrete mix with a prescribed *P_k_*, the probability of any point on the 2D cross-section lying on the diameter of *D* < *D_i_* for the different sieve diameters *D_i_* can be calculated according to Equation (9).

If the probability density curve derived from Equation (9) is divided into several segments according to the sieve diameters, the area of aggregate with the diameter *D* ∈ [*D_i_*, *D_i_*_+1_] can be written as:

(10) $$A_{agg}(D_{i},D_{i+1})=P_{agg}\times A\times [P(D<D_{i+1})-P(D<D_{i})]/[P(D<D_{\max})-P(D<D_{\min})]$$

where *D_max_* and *D_min_* are the maximum and minimum diameters, respectively, *P_agg_* is the aggregate area percentage in the concrete model area A. After the preparation done above mentioned, we could use the random procedure of particle generation to produce aggregates whose sizes are in the range of *D* ∈ [*D_i_*, *D_i_*_+1_], until to reach the aggregate area of *A_agg_*. These aggregates just produced are stored as a library, which is the source of trial placement aggregates.

## 3. Detection and Identification of a Point in Polygon or Polyhedron

An overlap detection algorithm has to be able to evaluate points of contact between each pair of polygons in 2D or polyhedra in 3D. Here we handled the problem of judging the intersection of aggregate particles through computational geometry and computer graphics.

### 3.1. Point in General Polygon

Before elaborating the dot matrix method used to check the status of a trial placement of aggregate overlapped with the placed aggregates, we briefly introduced that probably the most used and effective algorithm for determining if a point *P* is inside a general polygon involves analyzing the intersections of the polygon and a ray whose origin is *P* and whose direction is coincided with the axis direction of X or Y in Cartesian coordinate system [34]. The determination of location of a point *P* if it is in the polygon is a salient part of the DMM we proposed. A common query in aggregate placements is to determine if a point is inside a placed aggregate (i.e., a polygon in 2D or polyhedron in 3D). As in graphics applications, many approaches that have been developed can be used to answer the query [34]. We discussed here the method that does not require preprocessing to create data structures that support fast queries. The advantage is that this method does require preprocessing by decomposing the polygon into trapezoids.

**Figure 4:**
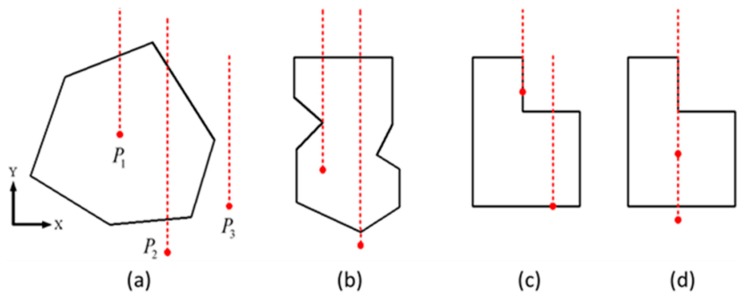
Point-in-polygon test by counting intersections of ray with polygon. (**a**) Only one intersection with the polygon, (**b**) ray through the vertex, (**c**) ray on the edge, and (**d**) ray coincided with the edge.

Generally, there are three kinds of the positional relationship between a point P and a general polygon, i.e., the point *P*, (a) outside the polygon or (b) on the edge of the polygon, or (c) inside the polygon. The principle of judgment is in a 2D case to count intersections of ray with polygon, in which the ray is along the direction of Y-axis. If the number of intersections is odd, the point is inside the polygon, otherwise, if there is no intersection or the number of intersections is even, the point is outside the polygon. For example, in Figure 4a, *P*_1_ holds only one intersection with the polygon, *P*_2_ holds two intersections with the polygon, but there is no intersection between *P*_3_ and the polygon. It is noted that the odd or even number of intersections of the ray with polygon is only a basic rule, but specifically, this rule may fail to be used to detect the intersection with a polygon such as ray through the vertex in Figure 4b, ray on the edge in Figure 4c, and ray coincided with the edge in Figure 4d.

**Figure 5:**
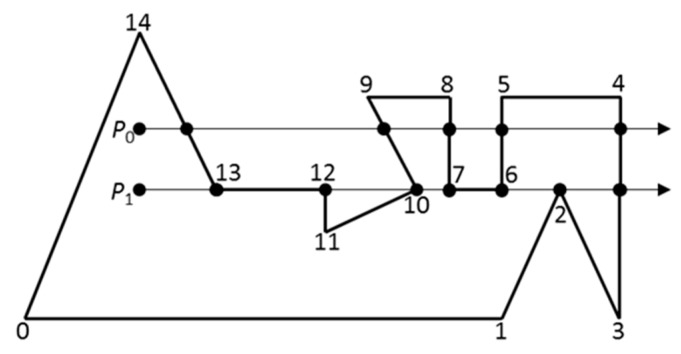
Point-in-polygon test by counting intersections of ray with polygon. The ray for point *P*_0_ only crosses edges transversely. The number of crossings is odd (5), so the point is inside the polygon. The ray for point *P*_1_ is more complex to analyze (redrawn according to [34]).

How to deal with the configurations aforementioned has been proposed respectively in [35] and [36]. The essential idea is that as shown in Figure 5, an edge is counted as a crossing of the ray with the polygon if one of the end points is strictly above the ray and the other end point is on or below the ray, e.g., edge <13, 14> [34]. Having this convention in mind, coincident edges are indeed not counted as crossing edges and certainly can be ignored, e.g., edge <12, 13>. Two edges above the ray that share a common vertex on the ray both count as crossings. If two edges below the ray share a common vertex on the ray, neither edge is counted, e.g., edge <6, 7>. If one edge is above and one edge is below the ray, both sharing a vertex on the ray, the edge above is counted but the edge below is not.

**Figure 6:**
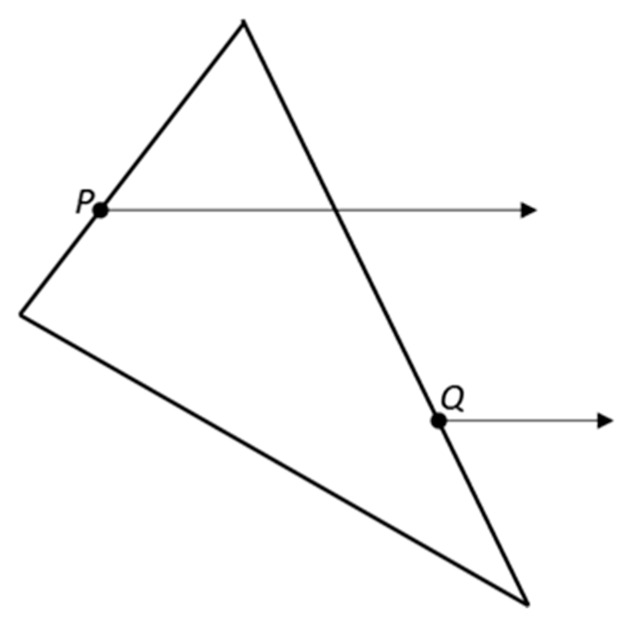
Points *P* on the ‘left’ edges of the polygon are classified as inside. Points *Q* on the ‘right’ edges of the polygon are classified as outside (redrawn according to [34]).

The point counting in the polygon aforementioned properly classifies points that are strictly inside or strictly outside the polygon. However, points on the boundary sometimes are classified as inside, sometimes as outside. In Figure 6, a triangle and two points *P* and *Q* on the boundary are indicated. The point *P* is classified as inside, but the point *Q* is classified as outside. We desired such behavior in an aggregate placement when two polygons share an edge. A point can only be in one polygon or the other, but not both. In aggregate placements where any edge point is required to be classified as inside, the present algorithm could trap the case when the intersection of an edge with the ray occurs exactly at the test point. These are detailed in [34]. Some other issues dealt with the configurational schemes can be also referred in [36,37]

### 3.2. Point in General Polyhedron

Similar to the 2D case, a standard query in aggregate placement processes is to determine if a point is inside a polyhedron. So, we continued to employ the idea in Section 3.1 to detect the overlapping between aggregates in 3D case [34]. Firstly, we needed to extend the point-in-polygon algorithm for a general polygon to three dimensions. In an obvious way, we partitioned the polyhedron space into a bounded inside zone and an unbounded outside region. Let a test point ***P*** be at the origin of a ray with the direction ***d***=(1,0,0), which is intersected with the faces of the polyhedron. It is noted that the number of intersections could be evaluated. If it is assumed that the ray only intersects the polyhedron at interior face points, the parity of the number of intersections characterizes inside from outside. If the parity is odd, the point is inside. Otherwise, the parity is even and the point is outside.

**Figure 7:**
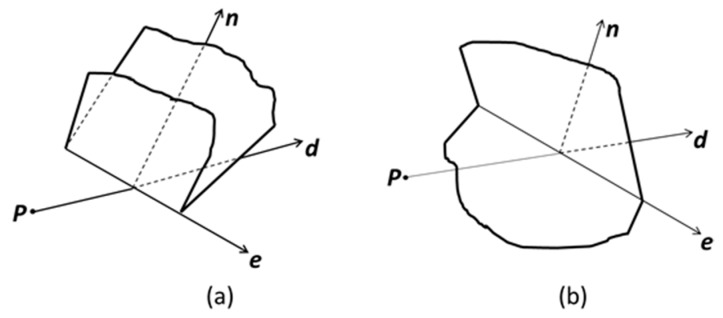
Illustration for two configurations for when the test ray ***P*** + *t**d*** intersects a shared edge ***e*** at an interior edge point, where ***n*** represents the normal direction of ***e*** × ***d***. (**a**) The faces are on the same side of the plane formed by the edge and the ray, while parity is not changed. (**b**) The faces are on opposite sides, thus parity is toggled.

However, when the ray intersects the polyhedron at vertices or interior edge points, the parity might be incorrectly evaluated. To avoid this mistake, we utilized the vertex-edge-face table to represent the polyhedron. The underlying algorithm performs an iteration over the faces of the polyhedron while each processed face is tagged that it was visited. If the ray intersects a face at an interior edge point, the adjacent face that shares the edge is immediately tagged as visited so that when it is visited later in the iteration, it is not tested for intersection. Furthermore, the parity will have to be adjusted based on the local configuration of the ray, the common edge, and the faces sharing that edge. These can be illustrated in Figure 7 where the local configuration is determined by a simple task of selecting two vertices. One comes from each face but not on the common edge, and at the same time, we evaluated on which side of the ray-edge plane they lie on. We again emphasized that the ray-intersections-edge situation is the analogy of the ray-intersections-vertex situation in the 2D case.

**Figure 8:**
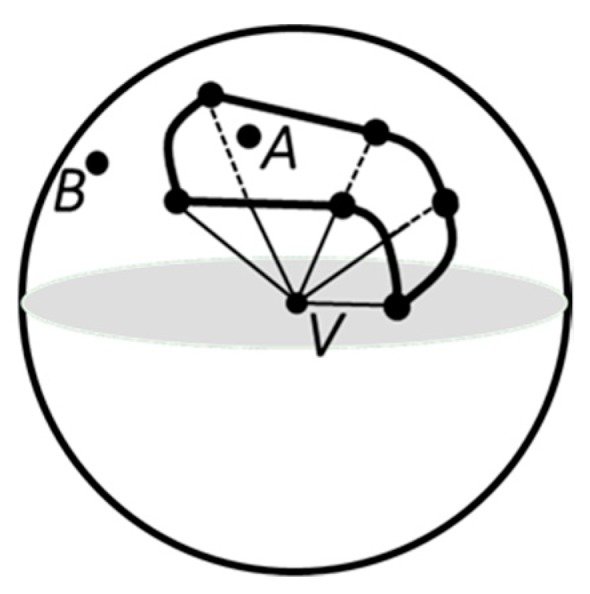
The spherical polygon indicated by the edges sharing a vertex *V* that the test ray intersects. If the point *A* corresponds to the ray direction, the ray interpenetrates the polyhedron. If the point *B* corresponds to the ray direction, the ray does not interpenetrate the polyhedron (redrawn according to [34]).

When the ray intersects a vertex ***V***, more complicated issue needs to be handled. That is to decide if the ray penetrates the polyhedron at *V* or if it just grazes the vertex so that locally the ray remains in the same zone. As an example, we allowed ***V*** = ***P*** + *t*_0_***d*** for some parameter *t*_0_. For a suitable small ε > 0, we needed to determine if the two open line segments corresponding to parameter intervals (*t*_0_ − ε) and (*t*_0_ + ε) are both inside or both outside, in which case the current parity is not changed, or one is inside and one is outside, in which case the current parity is toggled. We could imagine a very ruffled vertex whose adjacent faces form a triangle strip that wanders aimlessly through space locally at the vertex, probably making the issue appear to be hardly treated. If a unit sphere is centered at ***V*** and the edges sharing ***V*** are rescaled to be the unit length, the corresponding spherical points form a simple closed curve on the sphere, more precisely a piecewise-defined curve whose pieces are great circle arcs. The interior region bounded by that curve corresponds to the interior of the polyhedron at ***V***. The ray direction itself can be normalized and corresponds to a point on the sphere. The ray interpenetrates the polyhedron at ***V*** if and only if the corresponding sphere point is inside the spherical polygon implied by the edges sharing *V*. This is illustrated in Figure 8. An alternative to the fine-scale handling is to use a randomized approach, which is to generate random directions until a ray is found that only intersects faces at interior point. This can be referenced to [34] and among others.

## 4. The Dot Matrix Method of Aggregate Placement

After the shape and size of aggregate particles are determined, how to place them into a pre-defined empty container according to grading curves is not only to affect the density of concrete, but also to play a paramount role in the fracture processes. One of the essential tasks for virtual numerical concrete modeling is to tackle the placement of aggregates in concrete based upon the random distribution of grading particles. In the beginning, we created an empty container to represent a specimen, and then all the aggregates from the larger to the smaller were placed one after another into this container. In general, it started with the largest aggregates to be placed as it would be difficult to place them if they were processed at a later stage. There were three challenging tasks to be done as follows. (i) Check whether convex–concave profiles between placement particles are overlapping or not. This process usually requires knowledge of computational geometry. (ii) Commonly check one by one with all the aggregates that have been placed. However, to the best knowledge of the authors, there is no recognized most effective algorithm for detecting and identifying the overlapping of arbitrary-shape aggregates currently. (iii) As the amount of the already placed aggregates is increased, the try-placement aggregates may intersect with the placed ones, which are a high probability event. Further, the number of invalid try-placements leads to the subsequent inefficient overlap detection, which seriously influences the generation efficiency of mesostructure concrete material models.

**Figure 9:**
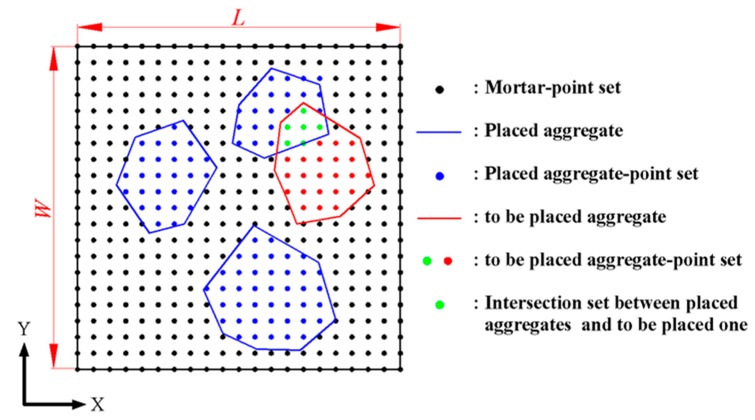
A placement procedure of aggregate by the dot matrix method proposed.

We developed a novel method for bypassing the disadvantage of the aggregate placement methods aforementioned. To give a framework of understanding, we took a two-dimensional polygon as an example to introduce some concepts that will be used later. The basic idea is to transform the overlap detection between polygons into the problem of whether there is any intersection between the point sets. Now let us take any rectangular concrete specimen (container), e.g., 100 mm × 100 mm, as an example to detail the placement procedure of aggregates, as shown in Figure 9. To conveniently understand the placement procedure, we regarded any rectangular container as a dot matrix. The distance between any neighbor two points was determined by a minimum size of particle to be placed. Mortar-point set (*setMor*), placed aggregates and their corresponding point set (*setAgg*), trial placement aggregates and their corresponding point set (*setTry*), and intersection set between placed aggregates and trial placement aggregates, are also defined in Figure 9, respectively. We assumed that the successful placement aggregates in the container were not allowed to be intersected. In other words, the mutual intersection set between these successful placement aggregate sets should be empty.

### 4.1. A Representation of Dot Matrix for Model Geometry

Let the length, width, and height of a model geometry (rectangular box or container) be *L*, *W*, and *H*, respectively. In the Cartesian coordinate system, the numbers of points in the geometry along with the directions of *x*, *y*, and *z* are *NumX*, *NumY*, and *NumZ*, respectively, as indicated in Figure 9 (to 2D case). The serial number of point is *N* ∈ (1, *NumX* × *NumY*) (to 3D case, *N* ∈ (1, *NumX* × *NumY* × *NumZ*), their order is in turn from the left-to-right to the bottom-to-top. These serial numbers are firstly stored in the mortar point set (*setMor*). After the dot matrix is created, three attributes are held for any point among the model. These are the serial number *N*, the row and column (*i*, *j*; to 3D case, (*i*, *j*, *k*)), and the coordinates (*x*, *y*; to 3D case, (*x*, *y*, *z*)). It is noted that if anyone among three attributes is known, the two others can be derived from it. For instance, from Equation (11), we could obtain the serial number *N* of point P from its row and column (*i*, *j*; to 3D case, (*i*, *j*, *k*)).

(11) $$\{\begin{array}{ll} N=(i-1)\times NumX+j, & \left(\mathrm{to}\;2D\;\mathrm{case}\right)\\ N=(k-1)\times (NumX\times NumY)+(i-1)\times NumX+j, & \left(\mathrm{to}\;3D\;\mathrm{case}\right) \end{array}$$

where *i* = 1, 2, …, *NumX*, *j* = 1, 2, …, *NumY*, *k* = 1,2, …, *NumZ*.

From Equations (12a) and (12b), the row and column (*i*, *j*; to 3D case, (*i, j, k*)) can also be represented in terms of the serial number *N* of point *P*,

(12a) $$\left\{ \begin{array}{l} i=ceil(N/NumX)\\ j=NumX-(i\times NumX-N)\;(\mathrm{to}\;2D\;\mathrm{case}) \end{array}\right.$$

(12b) $$\left\{ \begin{array}{l} k=ceil(N/(NumX\times NumY))\\ i=ceil(N-(k-1)\times (NumX\times NumY))/NumX\\ j=N-(k-1)\times (NumX\times NumY)-(i-1)\times NumX\;(\mathrm{to}\;3D\;\mathrm{case}) \end{array}\right.$$

where *ceil*() is the ceiling function, being the round up value.

Certainly, from Equation (13), the coordinates of point *P*(*x*, *y*) (or to 3D case, *P*(*x, y, z*)), can be obtained from its row and column (*i, j*; or to 3D case (*i, j, k*)).

(13) $$\left\{ \begin{array}{l} x=(j-1)\times L/(NumX-1)\\ y=(i-1)\times W/(NumY-1)\\ z=(k-1)\times H/(NumZ-1) \end{array}\right.$$

After the coordinates of point *P*(*x, y, z*) are determined, we could check if it is in the interior of a polygon or polyhedron (to be placed aggregate).

### 4.2. Aggregate Placement

Let point sets *setAgg* and *setTry* store the serial numbers of points within placed aggregates and the serial number of points within a trial placement aggregate, respectively. After the trial placement aggregates ordering from the larger one to the smaller one, we could place these aggregates into the dot matrix one after another from the larger one to the smaller one. Therefore, the times of try-placement are reduced, and the probability of try-placement successfulness is enhanced. Since all the trial placement aggregates are created from the same origin, the coordinates of the try-placement aggregates need to be translated into the dot matrix coordinate system during aggregate parking.

Firstly, we randomly took a serial number *N* from mortar set, *setMor*, so that its coordinates, (*x, y, z*), can be obtained from Equations (12a), (12b) and (13). Further, we translated the origin coordinate of a trial placement aggregate to the coordinate position to the serial number *N*. Next we checked if this placement was successful to meet two conditions: (a) the trial placement aggregate is inside the computational domain and (b) if the condition (a) is satisfied, the point set of *setTry* needs to be determined but not allowing intersection between *setTry* and *setAgg*.

If the two conditions above-mentioned are satisfied, this aggregate placement can be considered to be successful. Next, this *setTry* was copied into *setAgg*. Then the difference operation of the point set, (*setMor* *− setTry*), must be done to eliminate the successful placement aggregate previously from the mortar point set. Finally, the trial placement point set of *setTry* was emptied. However, if one of the two conditions above-mentioned is not satisfied, another point *N* needs to be taken from the mortar point set of *setMor*. The coordinates of point *N* also need to be determined. The point *N* was taken as the center of the to-be-placed aggregate and we again checked if the two conditions aforementioned were satisfied. Others need to be repeatedly done as did like the previous discussion until completing aggregate placement. A more detailed procedure of aggregate placement is given as follows.

Step 1. Read the container size, i.e., *L*, *W*, *H*, *NumX*, *NumY*, *NumZ*, and the aggregate volume fraction, *AggRatio*.

Step 2. (i) Generate a database of grading trial placement aggregates, and sort the aggregates in the database from the larger one to the smaller one; (ii) empty three point sets: *setMor*, *setAgg*, and *setTry*; and (iii) matrix the container domain, and store all the corresponding point serial numbers to *setMor*.

Step 3. Initialize *AggTotal* = 0.0 (i.e., assign an initial value of null to the area of all aggregates in the container), and update the total areas of the current aggregates when successfully placing.

Step 4. Take individual aggregate from the database as a trial placement one.

Step 5. Choose a point randomly from *setMor* and translate the origin of the trial placement aggregate in Step 4 to the position of this point.

Step 6. Check if the trial placement aggregate is properly in the computational domain. If the answer is yes, then go to Step 7, else if the answer is no, then go to Step 5.

Step 7. Determine for the point set of a trial placement aggregate, *setTry*.

Step 8. Check if there exists any intersection set between *setTry* and *setAgg*. If the answer is yes, then empty point set *setTry* and go to Step 5, else if the answer is no, then go to Step 9.

Step 9. (i) Store the data information of successfully placed aggregates, e.g., their vertex coordinates; (ii) add setTry into *setAgg*, next subtract *setTry* from *setMor*, and then empty *setTry*; and (iii) add the area (or volume) of aggregate into *AggTotal*.

Step 10. Check if *AggTotal* < (*AggRatio* × (*L* × *W* × *H*)) is satisfied, if the answer is yes, then go to Step 4, else if the answer is no, then the process of aggregate placements terminates.

From the process of a typical try-placement, we readily find that the position of the aggregate placement is in the mortar point set of *setMor*, in our DMM. In other words, the successful placement aggregates in *setAgg* have been moved into some regions. These regions were ever occupied by some mortar points in *setMor* so that at this moment the new mortar point set of *setMor* should be updated by *setMor* subtracting *setAgg*. Therefore, it is impossible to occupy the positions of the placed aggregates by the new consequent placement aggregates. Indeed, DMM dramatically enhances the probability of successful aggregate placement and reduces the number of placement attempts. Another merit of our DMM is that the complicated geometry evaluation is not involved in justifying the overlap between trial placement aggregate and placed ones in the container and instead arbitrary shape of aggregates including concave or convex shapes and uneven profiles can be processed. A most salient issue in the present method is how to determine the trial placement aggregate set of *setTry*, which will be treated in the later section. The overlapping judgment between aggregates is transformed into the issue of whether there is an intersection between the point sets, which makes the problem easier to understand and more convenient in the implementation of programming algorithms.

### 4.3. Determination of a Trial Placement Aggregate Set of setTry

**Figure 10:**
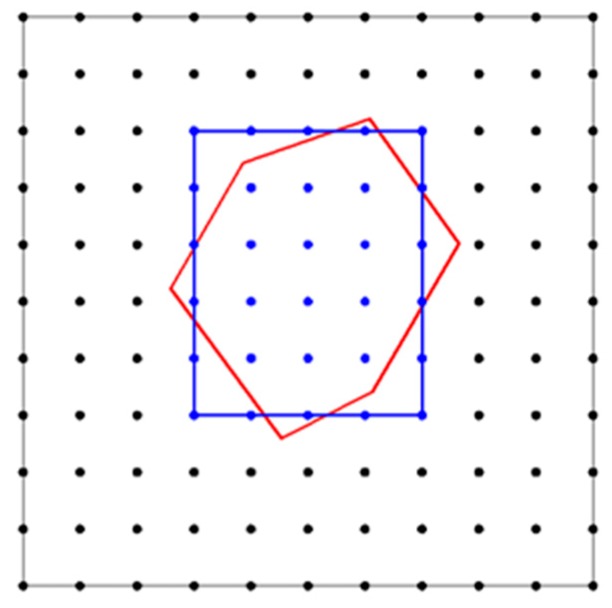
Illustration for a minimum rectangular box (the blue color) that contains all the points to be placed in the trial placement aggregate (area surrounded by the red color lines).

How to quickly locate the points inside the trial placement aggregate in a container largely determines the efficiency of aggregate parking. The easiest way is to traverse all the points in the mortar point set, *setMor*, and determine if each of the points is inside the trial placement aggregate. However, this method is extremely inefficient. One of the ideal methods should consider the points solely around a trial placement aggregate. Thus the approximately occupied region of the trial placement aggregate in the container can be determined. At the same time, the number of judgments for the point positions can be greatly reduced, and the calculation efficiency can be improved. The procedure we proposed was that a minimum rectangular box, which could at least contain all the points in a trial placement aggregate, was first determined, as indicated in Figure 10 (to 2D case). This box is determined by Equations (14a) and (14b),

(14a) $$\left\{ \begin{array}{l} Left=1+ceil(\min\_X\times (NumX-1)/L)\\ Right=1+floor(\max\_X\times (NumX-1)/L)\\ Bottom=1+ceil(\min\_Y\times (NumY-1)/W)\\ Top=1+floor(\max\_Y\times (NumY-1)/W) \end{array}\right.,\;\left(\mathrm{to}\;2D\;\mathrm{case}\right)$$

(14b) $$\left\{ \begin{array}{l} Left=1+ceil(\min\_X\times (NumX-1)/L)\\ Right=1+floor(\max\_X\times (NumX-1)/L)\\ Bottom=1+ceil(\min\_Y\times (NumY-1)/W)\\ Top=1+floor(\max\_Y\times (NumY-1)/W)\\ Back=1+ceil(\min\_Z\times (NumZ-1)/H)\\ Front=1+floor(\max\_Z\times (NumZ-1)/H) \end{array}\right.,\;\left(\mathrm{to}\;3D\;\mathrm{case}\right)$$

where *Left* and *Right* are the columns on the left and right boundaries of the box, respectively; *Bottom* and *Top* indicate the rows on the bottom and top boundaries of the box; *Back* and *Front* indicate the layers on the back and front boundaries of the box; *ceil*() and *floor*() give the corresponding round up value and round down value; min_*X*, max_*X*, min_*Y*, max_*Y*, min_*Z*, and max_*Z* are the corresponding minimum and maximum coordinates of the polygon vertices in the trial placement aggregate. From Equations (14a) and (14b), the range of the rows, columns, and layers of the points surrounded by the box in the dot matrix can be inferred, and the coordinates of each point are evaluated by Equation (13), and the positional relationship between the points and the polygon will be used to determine *setTry*, in which the points are all inside the polygon.

**Figure 11:**
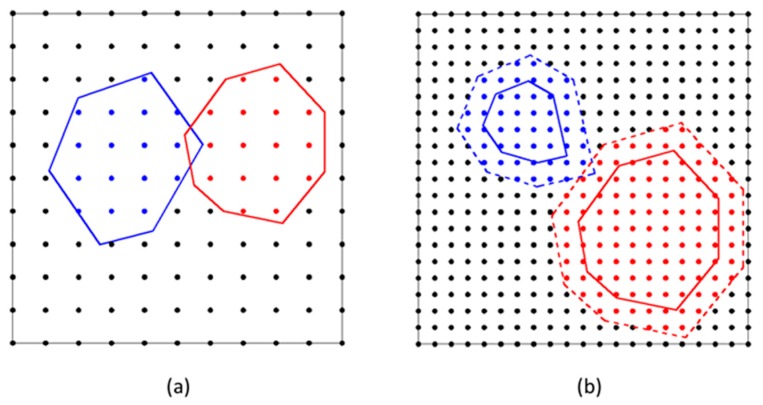
(**a**) In the procedure of placement aggregates, the geometrical overlap between placed aggregate (the red zone) and trial placement one (the blue zone) may occur potentially within the one unit of dot matrix, even though any intersection set between their point sets does not exist at all. (**b**) After detecting the status of intersection set between the points (the blue zone) surrounded by the virtual boundary of a trial placement and the points by the placed one (the red zone), the determination of the locations and vertexes in the trial placement aggregate is stored or not.

During the aggregate placement, the geometrical overlap between a try-placement aggregate and the already placed ones may occur potentially within the one unit of dot matrix (see in Figure 11a), even though there is not any intersection set between their point sets at all. To overcome the overlap, we appropriately zoomed in the profile of a trial placement aggregate as a virtual boundary. The point set surrounded by the virtual boundary was used to examine if it overlaps with the point set (*setAgg*) of the already placed aggregates. In detail, the length at the direction of the polar axis was increased by a segment, *T*/2, where *T* is the minimum value of allowance distance between aggregates in concrete. From our experience, this allowance distance *T* can be evaluated by Equation (15). Moreover, such parameter *T* introduced also benefits to avoid the singularity of the mesh shape when meshing later. After detecting the status of intersection set between the points surrounded by the virtual boundary of a try-placement and the points by the already placed ones, the determination of the locations and vertices in the trial placement aggregate was stored or not, as indicated in Figure 11b.

(15) $$T=4\times {\left[{\left(L/(NumX-1)\right)}^{2}+{\left(W/(NumY-1)\right)}^{2}+{\left(H/(NumZ-1)\right)}^{2}\right]}^{1/2}$$

We now needed to address how to realize the point set operations such as their intersection and difference. In general, there are two ways, such as self-developing algorithm and the functions in the standard template library (STL) to accomplish these tasks. The latter is used to efficiently implement such set operations in our dot matrix method because the functions *set*_*intersection*() and *set*_*difference*() in STL of the computer language C++ provide us with a solution to these problems. Fortunately, the two set functions in STL only have the linear time complexity *O*(*n*). Thus the executed efficiency of our procedure is only dependent on the point amount of dot matrix. Indeed, the larger the point number of dot matrix, the slower the running of placement aggregate procedure. As a guideline, *NumX* and *NumY* can be determined as follows:

(16) $$L/(NumX-1)=W/(NumY-1)=H/(NumZ-1)<D_{\min}/10$$

where *D_min_* represents the smallest size of aggregates in the study.

### 4.4. Generation of Irregular Stone and Rounded Stone Concrete Models

**Figure 12:**
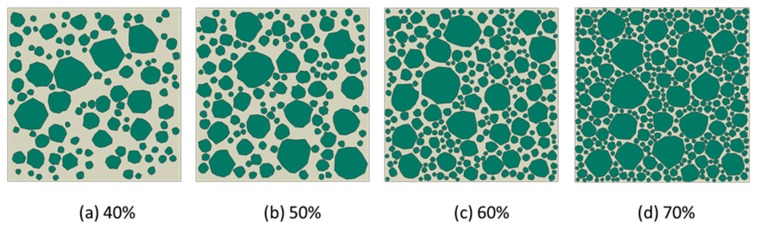
Irregular stone concrete models under different aggregate volume fractions with the scale factor between long and short axes, *f* = 1. (**a**) 40%; (**b**) 50%;(**c**) 60%;(**d**) 70%.

**Figure 13:**
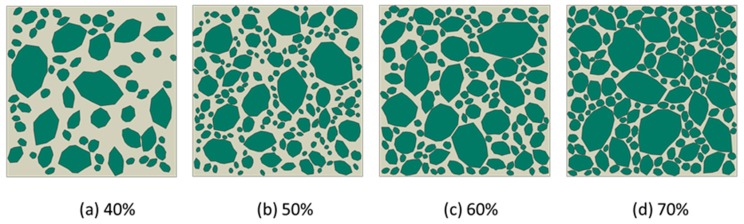
Elongated stone concrete models under different aggregate volume fractions with the scale factor between long and short axes, *f* ∈ [1,3]. (**a**) 40%; (**b**) 50%;(**c**) 60%;(**d**) 70%.

From the placement principle of numerical concrete constituents aforementioned, the concrete material models are given as in Figure 12, based on the irregular aggregate grading in concrete, while in Figure 13, the created concrete models were based on the elongated aggregate grading. Noted that in the present work, the sieve diameters were as follows, 2.36 mm, 4.75 mm, 9.5 mm, 16.0 mm, and 19.0 mm. Further, the minimum aggregate size was limited to 0.6 times the minimum sieve diameter, and the maximum aggregate size was 1.2 times the maximum sieve diameter.

**Figure 14:**
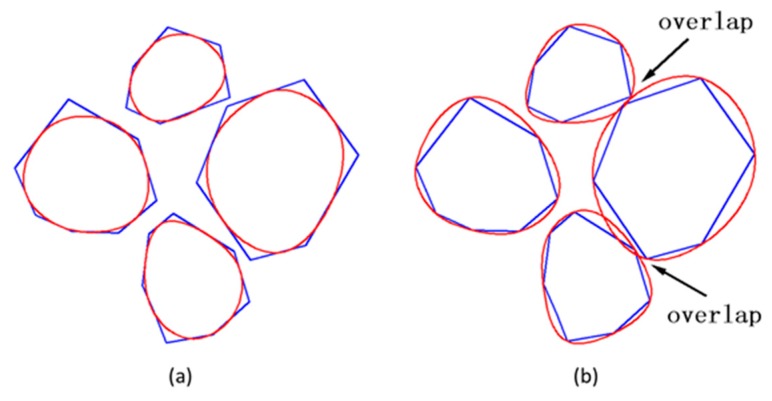
Illustration for the construction of gravel aggregates, (**a**) side midpoint as a spline control point, and (**b**) vertex as a spline control point. Among these, the red lines represent spline curves while the blue lines indicate aggregate boundaries.

**Figure 15:**
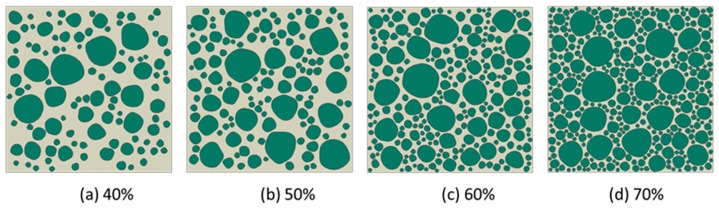
High-sphericity and well-rounded stone concrete models under different aggregate volume fractions, (**a**) 40%, (**b**) 50%, (**c**) 60%, and (**d**) 70%, respectively, with the scale factor between long and short axes, *f* = 1.

**Figure 16:**
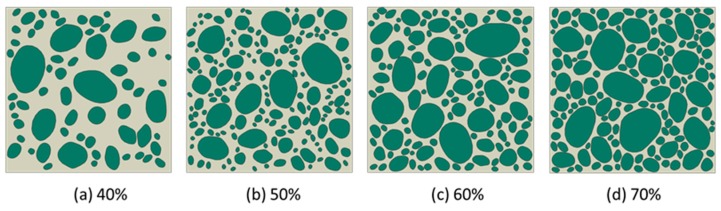
Low-sphericity and well-rounded stone concrete models under different aggregate volume fractions, (**a**) 40%, (**b**) 50%, (**c**) 60%, and (**d**) 70%, respectively, with the scale factor between long and short axes, *f* ∈ [1,3].

**Figure 17:**
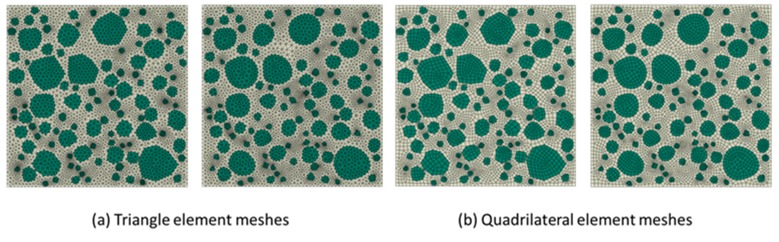
Typical element meshes for the irregular stone and rounded stone concrete models with the aggregate volume fraction 50%. (**a**) Triangle element meshes and (**b**) Quadrilateral element meshes.

The generation of rounded aggregates was based on the shape of crushed ones in the present work. The difference between the two fashions used to generate aggregates only was how to utilize the vertex data of polygon. For example, in Figure 14a, the construction of crushed aggregates was to connect the vertices of the polygons with straight lines. However, the construction of rounded aggregates uses the midpoint of each side of the polygon as a control point and connects the control points through the spline curves. In many meshing software systems such as Gmsh [38] and Abaqus [39], the script can provide a plotting function for the spline, as long as the coordinates of the control points given in turn. The rounded aggregate volume produced by this method will be slightly lower than the predetermined aggregate volume. This small difference is a side effect caused by the midpoint of each side of the polygon as a spline control point. However, if the vertex of the polygon is used as the spline control point, the final aggregate volume may not only be higher than the predetermined aggregate volume, but also cause mutual overlap between the aggregates, as shown in Figure 14b. Therefore, the former method was used to construct the shape of gravel aggregate in the present work. According to the construction principle of gravel aggregate, the numerical concrete models under different aggregate volume fractions are depicted as shown in Figure 15 and Figure 16. We could readily obtain the finite element meshes of such mesostructure concrete models by anyone of free or commercial FE meshing software systems such as Gmsh [38] and Abaqus [39], as given in Figure 17. It is noted that our proposed method could also be employed to provide the essential database for the discrete element methods when regarding placed aggregates as material particles.

### 4.5. Efficiency of Algorithms in the 2D Dot Matrix Method

**Figure 18:**
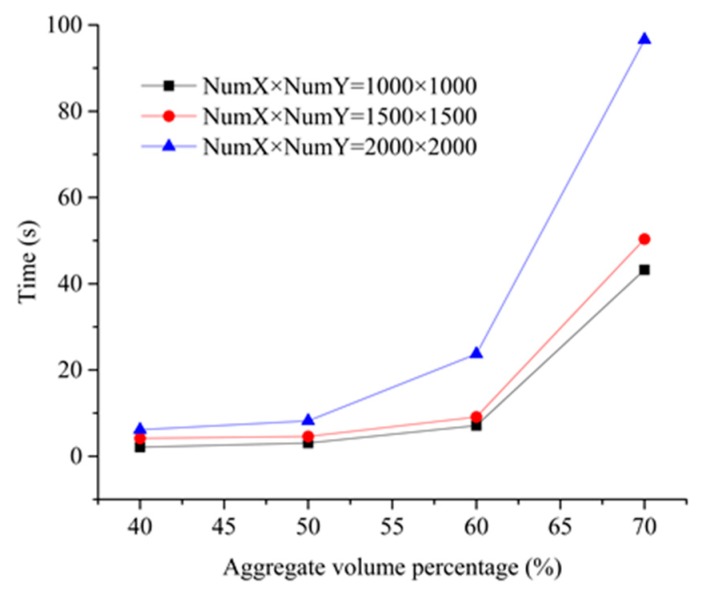
Execution time of aggregate placement with different aggregate volume fractions under different dot matrices.

To test the algorithm efficiency in the present DMM, we examined the time required to generate a numerical concrete model with different aggregate volume fractions using three dot matrices, as illustrated in Figure 18. The work was performed via the desk computer with Intel(R) Core(TM) i7-4810MQ CPU @ 2.80GHz. We found that as the number of dot matrix increased, the computational time of aggregate placement was enhanced. However, in the 2D case, a current desk computer with the memory of 8 GB was enough to process the laboratory scale model of interest. In general, the values of NumX and NumY satisfy the minimum requirement of Equation (16) to achieve the balance between calculation time and aggregate placement without the excessive pursuit of a larger number of the dot matrix.

### 4.6. Generation of 3D Rounded and Crushed Stone Concrete Models

**Figure 19:**
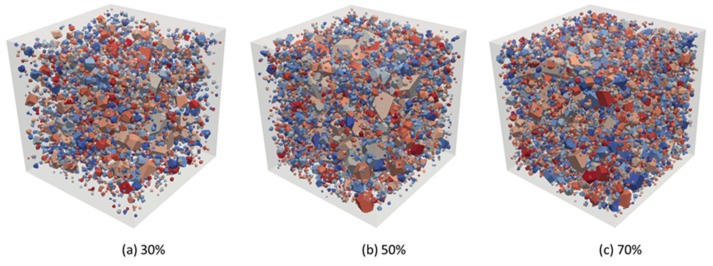
Numerical concrete models by irregular polyhedra, among which the aggregate volume fractions are (**a**) 30%, (**b**) 50%, and (**c**) 70%, respectively.

In the 3D case, the sieve diameters were as follows, 2.36 mm, 4.75 mm, 9.5 mm, 16.0 mm, 19.0 mm, 26.5 mm, and 31.5 mm. Similar to the 2D case, the minimum aggregate size was limited to 0.6 times the minimum sieve diameter, and the maximum aggregate size was 1.2 times the maximum sieve diameter. We used the 3D Voronoi cell library [33] to create the database of generating concrete composite material models. The irregular convex-shaped aggregates taken from the database according to the grading curve in Section 2.3 were placed into a pre-defined container using the present DMM, as shown in Figure 19. It is noted that in our DMM, we could create a numerical concrete model relying on grading curve and aggregate volume fraction, both accurately and flexibly.

**Figure 20:**
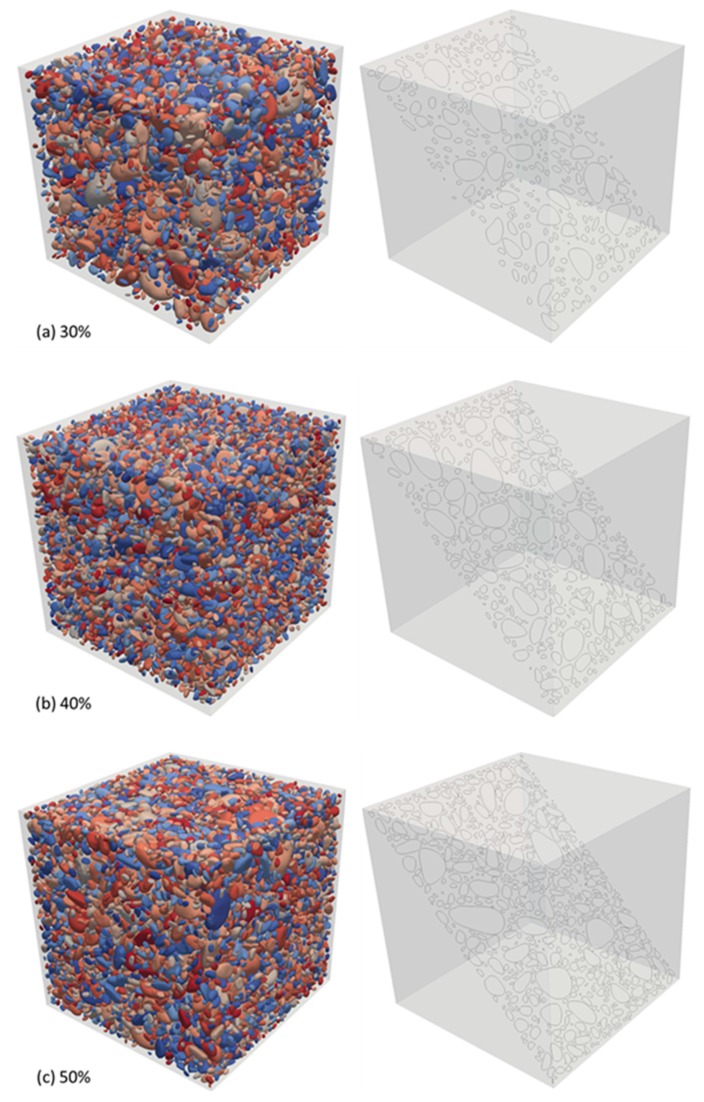
Numerical concrete models by rounded polyhedrons (where every right section represents the corresponding slice to help check if there is any overlapping aggregate conveniently), among which the aggregate volume fractions are (**a**) 30%, (**b**) 40%, and (**c**) 50%, respectively.

As the discussions in Section 1 and Section 2, the increasingly mature 3D laser scanning technology provides us with a tool to characterize the arbitrary surface morphology of aggregate particles. Thus we established the rounded aggregate database according to the grading curve above but the individual aggregate particles obtained by Artec 3D laser scanning equipment [32]. Consequently, we again employed DMM to construct the rounded aggregate composite models, as indicated in Figure 20. To conveniently observe the spatial distribution of the aggregate particles, we presented three typical slices that are also shown in Figure 20. Certainly, these slice results were not only to help understanding the mesolevel constitutions of the concrete models but also to verify and validate the present DMM’s applicability and efficiency. At the same time, we could check if there was any overlapping aggregate with help of slice at any position in the models to judge whether the concrete models by DMM were successful or not. It was emphasized here that since the output accuracy of the 3D laser scanning device was generally adjustable, we could consider the topography geometric data of a given aggregate from the viewpoint of the compromise in both compatibility and usability in accordance with the purpose and requirements of numerical concrete models. It was pointed out that the present 3D results were visualized by ParaView software (visited on 7 August 2019) [40].

**Figure 21:**
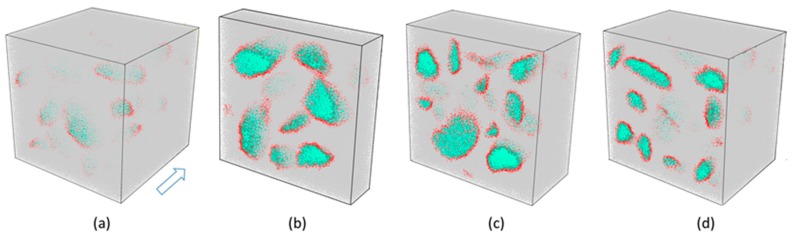
(**a**) A lattice model of numerical mesostructure concrete by the dot matrix method (DMM), (**b**)**,** (**c**), and (**d**) represents slices obtained from (**a**), respectively.

As an example of mesostructure concrete model by DMM, we employed DMM to construct the concrete geometry model with mesostructure. The aggregate volume fraction in the model was small to simplify the problem as well as to conveniently visualize the components. After these works, one of the simplest ways is that we could apply any mesh generation software to a geometry zone identified with the concrete model with the aggregate distribution to yield a great number of tetrahedron with mesh. We further utilized the surface coordinates provided by the aggregates in the mesostructure model to delimit where these aggregates locate in the mesh so that we could assign to the corresponding material properties with respect to aggregates, interface transformation zones, and mortar. Here we presented a lattice model by our DMM combined with Gmsh software [38], as shown in Figure 21. Among this lattice model, it is noted that the beams or rods between aggregate zones (represented by beams or rods with the aggregate properties) and mortar (also represented by beams or rods but with different properties to distinguish from the aggregates) are regarded as the interface beams or rods, which are characterized by interface models. Indeed we could establish reinforced concrete models by DMM, as did in [41,42,43], to investigate the behaviors of reinforced concrete beams. Furthermore, we could also construct beam-particle models to study cracking process in concrete by DMM combined with discrete element methods such as in [44,45]. These works will be done in the future.

The limit of the present DMM is that in the 3D case, a huge memory was sometimes required to deal with a large number of discrete integer and float variables in the study. For example, if a 3D dot matrix of concrete model is composed by the integer number of 1000 × 1000 × 1000, it at least needs approximately the memory of 7.45 GB for computers to process the primary data of interest. Therefore, further work is needed to develop a parallel computing method for DMM. Another future task that needs to be finished is how to deal with periodic material boundaries in the construction process of composite material models.

## 5. Conclusions

We proposed an innovative method of aggregate placement to generate concrete material models utilizing the principles of computational geometry. One of the advantages in the present DMM was that the examination of overlap between aggregates during the placement of aggregates realizes through the point set operations from the STL functions in the computer language C++ conveniently. This method also explicitly precludes complicated geometric calculations when judging the aggregate overlap during the aggregate placements. The most setting operations in DDM realized via integer arithmetic that the efficiency of our algorithms was usually higher in 3D aggregate placement applications. The present DMM is independent of the shapes of aggregate particles. If we applied the realistic configurations of aggregates through 3D laser scanning to create the database, the algorithm implementation of DDM was more flexible and convenient to generate mesostructure concrete models.

In the process of construction for aggregates, we introduced the scale factor f between long and short axes as a control parameter to better characterize the morphological characteristics of realistic aggregate particles. Another benefit of doing so is that we could take into account the random irregularness of an aggregate profile in the generation method. At the same time, we could also evaluate the characteristic quantities of concrete composite material models such as volume, area, and inertia tensor in the course of their establishment. The mesostructured concrete models in 2D and 3D cases created by the present DMM show that our procedures were feasible and robust. The 3D lattice model of concrete with a mesostructure was successfully provided by DMM combined with Gmsh software to investigate the cracking process of concrete under loads in the future. The limit of DMM is currently that sometimes a considerable memory is required in the 3D case to process a large number of discrete integer and float quantities of interest. The future task is to develop a parallel computing method for DMM and improve the present algorithm for adapting to periodic material boundaries in material models.

## References

[1] Alexander M., Mindess S. (2005). Aggregates in Concrete.

[2] Karavelić E., Nikolić M., Ibrahimbegovic A., Kurtović A. (2019). Concrete meso-scale model with full set of 3D failure modes with random distribution of aggregate and cement phase. Part I: Formulation and numerical implementation. Comp. Methods Appl. Mech. Eng..

[3] Garboczi E.J. (2002). Three-dimensional mathematical analysis of particle shape using X-ray tomography and spherical harmonics: Application to aggregates used in concrete. Cem. Concr. Res..

[4] Ren W.Y., Yang Z.J., Sharma R., Zhang C., Withers P. (2015). Two-dimensional X-ray CT image based meso-scale fracture modelling of concrete. Eng. Fract. Mech..

[5] Trawinski W., Tejchman J., Bobinski J. (2018). A three-dimensional meso-scale modelling of concrete fracture, based on cohesive elements and X-ray μCT images. Eng. Fract. Mech..

[6] Illerstrom A. (1998). A3-D Laser Technique for Size, Shape and Texture Analysis of Ballast. Master’s Thesis.

[7] Garboczi E.J., Cheok G.S., Stone W.C. (2006). Using LADAR to characterize the 3-D shape of aggregates: Preliminary results. Cem. Concr. Res..

[8] Latham J.-P., Munjiza A., Garcia X., Xiang J.S., Guises R. (2008). Three-dimensional particle shape acquisition and use of shape library for DEM and FEM/DEM simulation. Miner. Eng..

[9] Anochie-Boateng J.K., Komba J.J., Mvelase G.M. (2013). Three-dimensional laser scanning technique to quantify aggregate and ballast shape properties. Constr. Build. Mater..

[10] Wang X.F., Zhang M.Z., Jivkov A.P. (2016). Computational technology for analysis of 3D meso-structure effects on damage and failure of concrete. Int. J. Solid Struct..

[11] Ma H.F., Song L.Z., Xu W.X. (2018). A novel numerical scheme for random parameterized convex aggregate models with a high-volume fraction of aggregates in concrete-like granular materials. Comput. Struct..

[12] Lu Y., Garboczi E. (2014). Bridging the gap between random microstructure and 3D meshing. J. Comput. Civ. Eng..

[13] Qian Z., Garboczi E.J., Ye G., Schlangen E. (2016). Anm: A geometrical model for the composite structure of mortar and concrete using real-shape particles. Mater. Struct..

[14] He H. (2010). Computational Modelling of Particle Packing in Concrete. Ph.D. Thesis.

[15] Wang Z.M., Kwan A.K.H., Chan H.C. (1999). Mesoscopic study of concrete I: Generation of random aggregate structure and finite element mesh. Comput. Struct.

[16] Wittmann F.H., Roelfstra P.E., Sadouki H. (1985). Simulation and analysis of composite structures. Mater. Sci. Eng..

[17] Zhu Z.G., Chen H.S., Xu W.X., Lui L. (2014). Parking simulation of three-dimensional multi-sized star-shaped particles. Model. Simul. Mater. Sci. Eng..

[18] Beddow J.K., Meloy T.P. (1980). Testing and Characterization of Powders and Fine Particles.

[19] Zheng J.J., Guo Z.Q., Deng D., Stroeven P., Sluys L.J. (2011). ITZ volume fraction in concrete with spheroidal aggregate particles and application: Part I. numerical algorithm. Mag. Concr. Res..

[20] Thomas S., Lu Y., Garboczi E.J. (2016). Improved model for three-dimensional virtual concrete: Anm Model. J. Comput. Civ. Eng..

[21] Wriggers P., Moftah S.O. (2006). Mesoscale models for concrete: Homogenisation and damage behaviour. Finite Elem. Anal. Des..

[22] Qian Z. (2012). Multiscale Modeling of Fracture Processes in Cementitious Materials. Ph.D. Thesis.

[23] Torquato S. (2002). Random Heterogeneous Materials: Microstructure and Macroscopic Properties.

[24] Torquato S., Stillinger F.H. (2010). Jammed hard-particle packings: From Kepler to Bernal and beyond. Rev. Mod. Phys..

[25] Cooper D.W. (1988). Random-sequential-packing simulations in three dimensions for spheres. Phys. Rev. A.

[26] Garboczi E.J., Bulard J.W. (2017). 3D analytical mathematical models of random star-shape particles via a combination of X-ray computed microtomography and spherical harmonic analysis. Adv. Powder Technol..

[27] Garboczi E.J., Bullard J.W. (2013). Contact function, uniform-thickness shell volume, and convexity measure for 3D star-shaped random particles. Powder Technol..

[28] Ma H.F., Xu W.X., Li Y.C. (2016). Random aggregate model for mesoscopic structures and mechanical analysis of fully-graded concrete. Comput. Struct..

[29] Stroeven P., Hu J., Stroeven M. (2009). On the usefulness of discrete element computer modeling of particle packing for material characterization in concrete technology. Comput. Concr..

[30] Stroeven P., Le N.L., Sluys L.J., He H. (2012). Porosimetry by random node structuring in virtual concrete. Image Anal. Stereol..

[31] Nolan G.T., Kavanagh P.E. (1995). Random packing of nonspherical particles. Powder Technol..

[32] Science and Education 3D Models. https://www.artec3d.com/3d-models/science-and-education.

[33] Rycroft C. (2009). Voro++: A Three-Dimensional Voronoi Cell Library in C++.

[34] Schneider P.J., Eberly D.H. (2003). Geometric Tools for Computer Graphics.

[35] Haines E., Glassner A. (1989). Essential ray tracing algorithms. An Introduction to Ray Tracing.

[36] O’Rourke J. (1998). Computational Geometry in C.

[37] Eberly D.H. Polysolids and Boolean Operations. www.magic-software.com/Documentation/psolid.pdf1999.

[38] Geuzaine C., Remacle J.-F. (2009). Gmsh: A three-dimensional finite element mesh generator with built-in pre- and post-processing facilities. Int. J. Numer. Methods Eng..

[39] Dassault Systèmes Simulia Corp. (2014). Abaqus 6.14-1: Analysis User’s Manual.

[40] Kitware Inc. (2006). The VTK User’s Guide.

[41] Naser M.Z., Hawileh R.A. (2019). Predicting the response of continuous RC deep beams under varying levels of differential settlement. Front. Struct. Civ. Eng..

[42] Santarsiero G. (2018). FE modelling of the seismic behavior of wide beam-column joints strengthened with CFRP systems. Buildings.

[43] Hong K., Lee S., Yeon Y., Jung K. (2018). Flexural response of reinforced concrete beams strengthened with near surface mounted fe based shape memory alloy strips. Int. J. Concr. Struct. Mater..

[44] Vasaux M., Oliver-Leblond C., Richard B., Ragueneau F. (2016). Beam-particle approach to model crcking and energy dissipation in concrete: Identification strategy and validation. Cem. Concr. Compos..

[45] Grassl P., Bolander J. (2016). three-dimensional network model for coupling of fracture and mass transport in quasi-brittle geomaterials. Materials.

